# Supplementary material for: When RNA goes off script: ensuring transcript fidelity in transgene expression
Source: EMBO J. 2026 Mar 16;45(8):2420–32. doi: 10.1038/s44318-026-00733-z (PMC13083845; doi:10.1038/s44318-026-00733-z)
Supplement: Supplementary file 4 — Supplemental Information [file 44318_2026_733_MOESM4_ESM.docx]

# Supplemental Information

# Methods

## Cell culture

HEK293T (CRL-3216) were obtained from ATCC. Mycoplasma testing was performed quarterly using MycoAlert (Lonza LT07-318). Cell lines were maintained at 37 °C in 5% CO_2_ in Dulbecco's Modified Eagle Medium (DMEM; Gibco 11965126) supplemented with 10% fetal bovine serum (FBS; Gibco 26140079) and 1% Penicillin-Streptomycin-Glutamine (PSG; Gibco 10378016). Cells were passaged 3 times per week at 1:10 dilution using Dulbecco’s Phosphate Buffered Solution (DPBS; Gibco 14190144) and trypsin (Gibco 25200072).

For the transient transfection, cells were seeded in a 6-well plate at 750,000 cells/well overnight. 200 ng of each plasmid (11 plasmids in total, 2.2 μg) was pooled and mixed with 500 μL Opti-Mem (Gibco 31985070), after which 2.5 μL Plus Reagent and 8 μL Lipofectamine LTX (Invitrogen 15338100) were added. After a 10-minute incubation at room temperature (22 °C), the mixture was added dropwise to wells containing 1 mL Iscove's Modified Dulbecco's Medium (IMDM; Gibco 12440053). After 6 hours, the media was exchanged for DMEM with FBS and PSG.

## Design and cloning of splicing reporter library

## The split-mCherry reporter was synthesized by Twist Biosciences, where the intron 6 of *COQ8B* was inserted into the mCherry coding sequence. We made silent point mutations to mCherry to disrupt potential weak splice sites, to introduce ApaI and AgeI restriction sites for cloning the library, and to introduce a barcode close to the 5’ splice site. The barcode was placed at an amino acid sequence (Glu-Ala-Ser-Ser) which has many (2*4*6*6 = 288) potential codon encodings, allowing us to choose encodings which differed by at least three nucleotide positions. We chose to encode the barcode in the coding sequence so that it would be present regardless of whether or not the reporter had undergone splicing or not. We designed 10 test sequences, including epitope tags (HA, FLAG, and myc), and flexible linkers with 2 and 4 repeats, some of which were depleted for potential splicing signals. Test sequences were inserted mCherry backbone linearized by ApaI/AgeI by Gibson assembly. See Supplemental Table 2 for full plasmid sequences, barcodes, and individual descriptions for the tested sequences. All plasmids were verified by long-read sequencing (Quintara Bioscience).

## Splicing reporter library sequencing and analysis

RNA was collected from cells 24 hours after transfection using the PureLink RNA kit (Invitrogen 12183018A). RNA-seq libraries were prepared by Novogene Corporation with polyA enrichment and then sequenced as 150 bp PE reads on the Illumina NovaSeq X Plus.

Reads were aligned to hg38 with GRCh GTF annotation file (version 38.93) using the short-read alignment tool STAR^1^ (version 2.7.1a), using arguments “--outSAMtype BAM SortedByCoordinate --outSAMunmapped Within --genomeFastaFiles {all mCherry constructs.fa}” The aligned BAM files were indexed using samtools^2^ (version 1.1). We used a custom python python notebook to collect the primary alignments of read pairs which contained the barcode sequences (with no mismatches allowed) using pysam (version 0.16.0.1). Reads were then binned according to whether they were unspliced, spliced at the expected *COQ8B* 5’ and 3’ splice sites, or aberrantly spliced at sites involving the test sequence. Sashimi plots were prepared with ggsashimi^3^ (version 1.1.5). Raw RNA-seq data was uploaded to the NCBI Sequence Read Archive, accession number PRJNA1256814.

## Codon optimization

To codon optimize, we chose the most common human encoding for each amino acid from the Kazusa database^4^. We selected potential amino acid sequences likely to create 5’ and 3’ splice sites (e.g., QVS and PPPQ) by manual examination of codon optimized encodings and comparison with splicing signals.

## Splice site scoring and protein tag design

MaxEnt scores for 5’ and 3’ splice sites were calculated using the MaxEntScan web-server (<http://hollywood.mit.edu/burgelab/maxent/Xmaxentscan_scoreseq.html>^5^), or using maxentpy (https://github.com/kepbod/maxentpy/), a python wrapper for the same model. Transcriptome-wide analysis was performed using a custom python script which calculated the 5’ and 3’ MaxEnt score for exons in hg38. We did not control for exons processed by the minor spliceosome, which account for less than 1% of exons and can have different splicing signals.

For analysis of epitope tags, we generated 10^6^ random encodings of each tag. We used every possible nucleotide encoding of the amino acid sequence, plus two ‘N’ bases at the start and end of the encoding (to account for potential surrounding sequence context). To generate random linkers, we generated 10^6^ random encodings of (GGGGS)_N_ sequences for N=1-5 using every possible nucleotide encoding for Gly and Ser. In both cases, we moved a 9 base sliding window (for 5’ sites) or 23-base window (for 3’ sites) across the entire sequence and scored each window using the MaxEnt model as previously described. We took the highest scoring window across the sequence as the result for that particular sequence.

One encoding for the V5 epitope tag, previously reported to harbor a 5’ splice site, DNA sequence 5'-GGTAAGCCTATCCCTAACCCTCTCCTCGGTCTCGATTCTACG-3', is included in Snapgene’s list of common sequence features (version 8.0.3), and in at least 47 research articles (found by searching Google Scholar for the DNA sequence). Similarly, an encoding for the HA tag which we found can provide a 3’ splice site, DNA sequence 5'-TACCCATACGATGTTCCAGATTACGCT-3', is present in “empty” vectors on the Addgene plasmid repository (found by manual inspection) and in at least 44 research articles. Prevalence of these sequences in research articles is provided for broad commentary, and we did not control for articles where these sequences were employed in prokaryotic systems (which lack RNA splicing) or in lower eukaryotes (e.g., *S.cerevisiae*, which has more conserved splicing signals), or for articles where these tags are used without being explicitly reported in the main text.

To generate flexible linkers that lack predicted splicing signals, we generated 10^6^ random sequences for each, selecting encodings that minimized GC content and had MaxEnt scores < 0. For epitope tags, we introduced single-codon mutations to disrupt potential splice sites.

# **References**

1. Dobin, A., Davis, C.A., Schlesinger, F., Drenkow, J., Zaleski, C., Jha, S., Batut, P., Chaisson, M., and Gingeras, T.R. (2013). STAR: ultrafast universal RNA-seq aligner. Bioinformatics *29*, 15–21. https://doi.org/10.1093/bioinformatics/bts635.

2. Danecek, P., Bonfield, J.K., Liddle, J., Marshall, J., Ohan, V., Pollard, M.O., Whitwham, A., Keane, T., McCarthy, S.A., Davies, R.M., et al. (2021). Twelve years of SAMtools and BCFtools. Gigascience *10*, giab008. https://doi.org/10.1093/gigascience/giab008.

3. Garrido-Martín, D., Palumbo, E., Guigó, R., and Breschi, A. (2018). ggsashimi: Sashimi plot revised for browser- and annotation-independent splicing visualization. PLoS Comput. Biol. *14*, e1006360. https://doi.org/10.1371/journal.pcbi.1006360.

4. Nakamura, Y., Gojobori, T., and Ikemura, T. (2000). Codon usage tabulated from international DNA sequence databases: status for the year 2000. Nucleic Acids Res *28*, 292. https://doi.org/10.1093/nar/28.1.292.

5. Yeo, G., and Burge, C.B. (2004). Maximum Entropy Modeling of Short Sequence Motifs with Applications to RNA Splicing Signals. J. Comput. Biol. *11*, 2–3.
